# Perspectives of Indian Gastroenterologists and Hepatologists on Nonalcoholic Fatty Liver Disease Diagnosis and Management: Insights From the Nationwide Web-Based Cross-Sectional DRIVE Survey

**DOI:** 10.2196/75138

**Published:** 2026-03-02

**Authors:** Anil Arora, Yogesh Garje, Shagupta Shaikh, Shruti Dharmadhikari, Chintan Khandhedia, Neeraj Markandeywar, Amey Mane, Suyog C Mehta, Ashish Kumar

**Affiliations:** 1 Institute of Liver, Gastroenterology and Pancreaticobiliary Sciences Sir Ganga Ram Hospital, Rajinder Nagar New Delhi India; 2 Medical Affairs & Clinical Research Sun Pharma Laboratories Limited Mumbai, Maharashtra India

**Keywords:** nonalcoholic fatty liver disease, NAFLD, nonalcoholic steatohepatitis, NASH, gastroenterology, elastography

## Abstract

**Background:**

Nonalcoholic fatty liver disease (NAFLD) and its progressive form, nonalcoholic steatohepatitis (NASH), represent an increasing clinical and public health burden in India. Despite their high prevalence, there are limited data on their diagnostic and management approaches among Indian health care providers. Real-world evidence on how Indian gastroenterologists and hepatologists diagnose and manage these conditions remains limited.

**Objective:**

This study aimed to understand the current disease perspectives, diagnostic modalities, and management practices for NAFLD and NASH among Indian hepatologists and gastroenterologists.

**Methods:**

A nationwide, web-based cross-sectional survey was conducted online between May 2023 and July 2023 among practicing gastroenterologists and hepatologists from health care setups, clinics, and hospitals located across India. The structured, self-administered questionnaire included 34 items covering 3 domains: disease perspectives (n=16), diagnostic modalities (n=4), and management strategies (n=14). Descriptive statistics were used to summarize responses as counts and percentages.

**Results:**

A total of 609 physicians completed the online survey (gastroenterologists: n=556, 91.3%; hepatologists: n=53, 8.7%). For 336 (55.2%) physicians, NAFLD accounted for 25% to 50% of the patients consulted per month, and 220 (36.1%) physicians reported that 10% to 20% of patients with NAFLD had NASH. Obesity (n=583, 95.7%) and diabetes (n=579, 95.1%) were cited as leading risk factors for NAFLD. Transient elastography was the diagnostic tool preferred by 558 (91.6%) physicians, followed by NAFLD fibrosis score (n=378, 62.1%) and Fibrosis-4 score (n=356, 58.5%); only 154 (25.3%) physicians used liver biopsy. For treatment, 414 (68%) physicians managed patients using pharmacotherapy and dietary and lifestyle modifications, while 195 (32%) relied on lifestyle modification alone. Antioxidant vitamins (n=543, 89.2%) and saroglitazar (n=522, 85.7%) were the most frequently prescribed therapies. The main barriers to optimal NASH management reported were lack of patient awareness (n=466, 76.5%) and limited availability of effective pharmacological options (n=303, 49.8%).

**Conclusions:**

This large, nationwide survey highlights that NAFLD and NASH constitute a major part of gastroenterology and hepatology practice in India. Although transient elastography and pharmacological agents such as saroglitazar and vitamin E are widely used, considerable heterogeneity exists in diagnostic and management approaches. The lack of patient awareness and effective treatment options remain the major hurdles in managing NAFLD and NASH. These findings underscore the need for the wider implementation of existing India-specific consensus recommendations, continued physician education, and future research focusing on tailored interventions in the management of NAFLD and NASH for the Indian population.

## Introduction

Nonalcoholic fatty liver disease (NAFLD) is the most prevalent cause of chronic liver disease that encompasses a broad spectrum of clinical liver pathologies, including simple steatosis and nonalcoholic steatohepatitis (NASH), which could advance to fibrosis and cirrhosis, and may lead to liver failure or hepatocellular carcinoma (HCC) [1]. The worldwide prevalence of NAFLD is estimated to be 24% to 30% [2,3]. However, estimates for India vary and range from 9% to 53%. In India, NAFLD and NASH have been identified as predominant causes of cirrhosis, HCC, and liver transplantation [4]. A total of 20% of patients with NASH were reported to develop cirrhosis, and 30% to 40% of the patients with NASH cirrhosis died from liver-related causes [5,6].

In June 2023, an international consensus panel recommended revising the nomenclature of NAFLD and NASH to *metabolic dysfunction–associated steatotic liver disease (MASLD)* and *metabolic dysfunction–associated steatohepatitis (MASH)* to better reflect the underlying metabolic basis of the diseases [7,8]. However, the Indian National Association for Study of the Liver (INASL; 2023) continues to recommend the use of NAFLD or NASH terminology in India until broader consensus and implementation are achieved [9]. Importantly, INASL cautions that the new nomenclature “may create confusion not only among hepatologists but also among physicians and non-hepatologists who manage these patients or conduct research in this area.”

Various comorbidities have been reported among patients with NAFLD and NASH, such as diabetes, obesity, and hypertension [10]. NASH affects up to 30% of patients with obesity, which poses a serious risk to their liver and cardiovascular health [11]. Although various diagnostic strategies are available for the diagnosis of NAFLD and NASH in hepatology and gastroenterology practices, it is estimated that fewer than 20% of patients with NAFLD have been diagnosed [12,13]. In addition, among the diagnosed patients, very few are referred to hepatologists and gastroenterologists [14]. The mainstay of NAFLD treatment is lifestyle changes, including weight loss and physical exercise; however, many patients with NAFLD fail to lose weight [15,16].

The growing prevalence and limited number of approved medications for NAFLD and NASH remain a challenge in the management of these conditions [17]. In addition, long-term data on improvement in fibrosis with the existing therapies are lacking [18]. Therefore, it becomes imperative to understand the challenges faced by hepatologists and gastroenterologists in the diagnosis and management of NAFLD.

In a recent global survey, 68% of physicians reported lack of awareness as the most common reason for the limited screening of NAFLD, which can worsen the burden of NAFLD in the future [19]. The major barriers to NAFLD management include the lack of Indian data on the management of patients with NAFLD, low screening rates of vulnerable populations, and the paucity of suitable treatment options [9,17].

Therefore, to understand real-world perspectives of Indian hepatologists and gastroenterologists on NAFLD and NASH, a cross-sectional questionnaire-based disease perspective (DRIVE) survey was carried out between May 2023 and July 2023. As this survey was conducted *before* the global shift to MASLD and MASH terminology (June 2023), the survey questionnaire continued to use the NAFLD and NASH terms; therefore, these terms are retained throughout this paper for accuracy and consistency in reporting survey data.

This survey sought to capture the current clinical practices and perspectives on NAFLD and NASH management across India. The increasing global burden of metabolic liver disease and the variability in health care infrastructure and clinical practices across countries present significant challenges to standardized care. Evaluating diagnostic and therapeutic strategies from large-population settings such as India—where MASLD prevalence is rising rapidly—can provide critical insights, which can offer valuable lessons to other low- and middle-income countries facing similar implementation barriers [7,8].

## Methods

### Participants and Procedures

This web-based, cross-sectional survey was conducted online between May 15, 2023, and July 31, 2023, among practicing gastroenterologists and hepatologists across India to understand their perspectives on NAFLD and NASH. Eligible participants included practicing gastroenterologists and hepatologists working in various health care setups such as multispecialty hospitals, clinics, nursing homes, and government hospitals or medical colleges across the country who voluntarily agreed to participate in this survey. Invitations to participate were distributed electronically. Details on the number of invitations distributed and the source of the contact list were not recorded. The online survey questionnaire link was distributed electronically (via email), and gastroenterologists who provided a full response to the survey were included (N=609) in the analysis.

### Survey Questionnaire

A predefined, structured, and self-administered questionnaire was developed by the study investigators based on national (INASL) [9] and international clinical practice guidelines for NAFLD and NASH management [7,20]. The questionnaire included 34 questions distributed across 3 domains: disease perspectives (16 items), diagnostic modalities (4 items), and management strategies (14 items). Items were reviewed by hepatology experts to ensure relevance and clarity. Both closed-ended questions with predefined response options and open-ended questions for additional comments were included.

The “Disease Perspectives” section included 16 questions on the monthly NAFLD caseload, the percentage of patients with NAFLD who had NASH, biopsy rates among patients with NASH, common fibrosis stages at presentation, the likelihood of progression to cirrhosis within 5 years, and actions taken upon incidental NAFLD detection. It also covered risk factors, comorbidities, and symptoms commonly associated with NAFLD.

The “Diagnostic Modalities” section encompassed 4 questions focused on recommended tests for NAFLD diagnosis, additional laboratory data considered, scoring systems used, and indications for liver biopsies.

Finally, 14 questions in the “Management Strategies” section included referral patterns to dietitians, commonly prescribed medications, follow-up frequencies, monitoring methods, guidelines followed, challenges in managing NASH, and anticipated future treatment options. The pharmacotherapy domain of the questionnaire included the following response options: vitamin E or antioxidant formulations, saroglitazar, pioglitazone, and a separate category labelled “hepatoprotective agents,” which referred to ursodeoxycholic acid (UDCA), silymarin, and related antioxidant or herbal formulations commonly used in India [14,19].

The questionnaire included both open- and closed-ended questions to capture detailed insights. Demographic data on participants’ practice settings, clinical experience, the number of patients with NAFLD consulted within a given time frame, and specialty referral patterns for patients with NAFLD were also collected (Multimedia Appendix 1).

### Reporting Standards

This survey is reported in accordance with the CHERRIES (Checklist for Reporting Results of Internet E-Surveys) guidelines (Multimedia Appendix 2).

### Statistical Analysis

Survey data were analyzed using descriptive statistics, with results summarized as frequencies and percentages to illustrate the distribution of responses across different categories. Responses to open-ended questions were also summarized descriptively to identify common themes. Summary statistics were used for numerical variables wherever applicable. All analyses were conducted using SPSS software (version 16.0; IBM Corp).

### Ethical Considerations

This survey was reviewed and approved by the Royal Pune Independent Ethics Committee, Pune, Maharashtra, India (RPIEC110123; dated January 10, 2023). This was a physician-based survey with no involvement of patients or collection of patient-identifiable data. Hence, patient informed consent was not applicable.

Participation in this survey was voluntary and anonymous. Before accessing the survey, each participant was presented with an electronic informed consent form describing the survey objectives, the confidentiality of responses, the voluntary nature of participation, and the right to withdraw before final submission. Only those who provided electronic consent could proceed to complete the questionnaire. No personal identifiers were collected, and data were analyzed in aggregate.

No monetary or nonmonetary compensation was provided to the participants.

## Results

### Survey Participants’ Demographics

A total of 609 physicians (gastroenterologists: n=556, 91.3%; hepatologists: n=53, 8.7%) participated in this survey from various centers across India. All the participating physicians had completed the survey in full. Most of the participating physicians were male individuals (n=574, 94.3%). In this survey, most physicians (n=213, 35%) had 6 to 10 years of experience in clinical practice. Overall, 341 (56%) physicians predominantly practiced in multispecialty hospitals, while 120 (19.7%) worked in mixed settings (Table 1).

**Table 1:** Demographic details of the participants (N=609).

Variable			Participants, n (%)
**Sex**			
	Male	574 (94.3)	
	Female	35 (5.7)	
	Intersex	0 (0)	
**Duration of clinical practice (y)**			
	<5	149 (24.5)	
	6-10	213 (35)	
	11-20	178 (29.2)	
	>20	69 (11.3)	
**Predominant type of practice**			
	Multispecialty hospital	341 (56)	
	Mixed	120 (19.7)	
	Clinic or nursing home	95 (15.6)	
	Government hospital or medical college	53 (8.7)	

### Disease Perspective

Of the 609 physicians, 336 (55.2%) reported that the proportion of patients with NAFLD out of total patients examined each month was 25% to 50%. Furthermore, 220 (36.1%) physicians responded that 10% to 20% of their patients with NAFLD had NASH, and 523 (85.9%) physicians reported that less than 5% of their patients with NASH underwent biopsy. Patients with NAFLD presented to their physicians at varying stages of fibrosis. Most physicians (n=319, 52.4%) reported that majority of their patients with NAFLD first presented at stage 2 of fibrosis, while 204 (33.5%) physicians reported that 5% to 10% of patients with NASH progressed to cirrhosis over the course of 5 years.

Consulting physicians (n=491, 80.6%) were the most common specialties that referred patients with NAFLD to gastroenterologists and hepatologists (Table 2). According to 227 (37.3%) of the physicians, 25% to 50% of their patients with NAFLD were diagnosed incidentally. Among the patients with incidentally diagnosed NAFLD, various actions undertaken by physicians included investigation for the presence of metabolic alterations and the absence of other liver diseases (n=426, 70%), assessment for cardiovascular risk and liver disease (n=346, 56.8%), assessment for the risk of metabolic alterations and liver function with a noninvasive scoring system (n=499, 81.9%), investigation for presence of alcohol consumption (n=394, 64.7%), advice on lifestyle modification and follow-up (n=487, 80%), and initiation of pharmacotherapy (n=145, 23.8%), as reported by physicians.

Most physicians considered obesity (n=583, 95.7%) and diabetes mellitus (n=579, 95.1%) as the risk factors for NAFLD (Table 3). A total of 295 (48.4%), 149 (24.5%), and 300 (49.3%) physicians reported that 20% to 40% of their patients with NAFLD had diabetes, obesity, and dyslipidemia, respectively.

Of the 609 physicians, 331 (54.3%) reported that 10% to 30% of their patients with NAFLD required a multispecialty management approach. The proportion of lean NASH diagnosed among all patients with NASH per year was 3% to 5%, as reported by 224 (36.8%) physicians. Approximately 370 (60.8%) physicians recommended screening the family members of patients with NAFLD. Most physicians (n=345, 56.7%) reported that fewer than 10% of their patients with NAFLD were symptomatic. The most common symptoms reported to physicians by their patients with NAFLD were right upper quadrant pain (n=425, 69.8%), fatigue or tiredness (n=421, 69.1%), a feeling of fullness (n=286, 47%), and bloating (n=270, 44.3%; Table 4).

**Table 2:** Predominant specialties referring patients with nonalcoholic fatty liver disease to gastroenterologists or hepatologists (N=609).

Referring specialties	Survey responses, n (%)
Consulting physician	491 (80.6)
Endocrinologist	85 (14)
Cardiologist	16 (2.6)
General practitioner	7 (1.1)
Surgeons	3 (0.5)
Gynecologists	2 (0.3)
Others	5 (0.8)

**Table 3:** Risk factors for nonalcoholic fatty liver disease (NAFLD) as reported by survey participants.

Risk factor	Respondents, n (%)
Obesity	583 (95.7)
Diabetes	579 (95.1)
Dyslipidemia	547 (89.8)
Hypothyroidism	381 (62.6)
Polycystic ovary syndrome	367 (60.3)
Hypertension	335 (55)
Sleep apnea	281 (46.1)
Ischemic heart disease	189 (31)

**Table 4:** Symptoms commonly reported by patients with nonalcoholic fatty liver disease (NAFLD) as reported by survey participants (N=609).

Symptoms commonly reported by patients with NAFLD	Respondents, n (%)
Right upper quadrant pain	425 (69.8)
Fatigue or tiredness	421 (69.1)
A feeling of fullness	286 (47)
Bloating	270 (44.3)
Weakness	184 (30.2)
Asymptomatic	4 (0.7)
Other symptoms^a^	9 (1.5)

^a^Other symptoms include gastroesophageal reflux disease, incidental detection, incomplete evacuation of bowel, lethargy, loss of appetite, low mood, poor appetite, sense of indigestion, discomfort or pain after meals, and other complaints.

### Diagnostic Modalities

**Figure 1:**
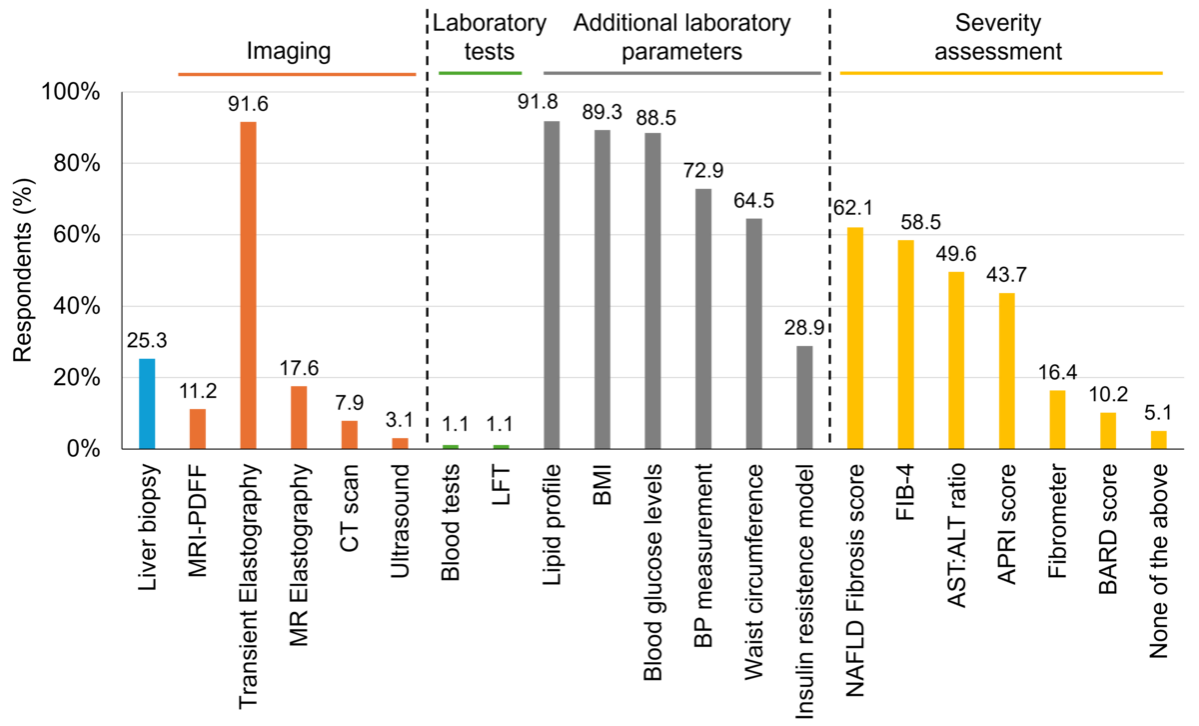
Summary of the clinicians’ preference for various diagnostic modalities to diagnose and differentiate between nonalcoholic fatty liver disease (NAFLD) and its subtypes in patients. ALT: alanine aminotransferase; APRI: aspartate aminotransferase-to-platelet ratio index; AST: aspartate aminotransferase; BARD score: BMI, AST:ALT ratio, and diabetes mellitus; BP: blood pressure; CT: computed tomography; FIB-4: Fibrosis-4; HOMA-IR: homeostatic model assessment of insulin resistance; LFT: liver function test; MR: magnetic resonance; MRI-PDFF: magnetic resonance imaging–proton density fat fraction.

For the diagnosis of NAFLD, most physicians preferred transient elastography (TE; n=558, 91.6%) followed by liver biopsy (n=154, 25.3%), magnetic resonance elastography (n=107, 17.6%), and magnetic resonance imaging–proton density fat fraction (n=68, 11.2%), computed tomography scan (n=48, 7.9%), and ultrasound (n=19, 3.1%). Additional tests or assessments routinely advised in patients diagnosed with NAFLD were lipid profile, BMI, blood glucose levels, and so on. To assess the severity of NAFLD, scores such as the NAFLD fibrosis score, Fibrosis-4 (FIB-4) score, aspartate aminotransferase-to-alanine aminotransferase ratio, and aspartate aminotransferase-to-platelet ratio index score were used (Figure 1). A total of 286 (47%) of the physicians reported that the main intent of advising a biopsy in patients with NAFLD was to determine the stage of fibrosis for prognostic purposes, while 236 (38.8%) of the physicians advised biopsy to differentiate NASH from steatosis for diagnostic purposes.

### Management Strategies

Of the 609 physicians, pharmacotherapy, along with dietary and lifestyle modifications, was prescribed by 414 (68%) physicians for the management of patients with NAFLD, while 195 (32%) physicians used only dietary and lifestyle modifications to manage their patients. It was reported that more than 30% of the patients were referred to a dietitian for dietary management by 278 (45.6%) physicians. A total of 499 (81.9%) physicians allowed no alcohol consumption for patients with NASH, and 110 (18.1%) physicians allowed 10 to 30 g of alcohol per day.

Antioxidant vitamins were the most often prescribed drugs (n=543, 89.2%) for the treatment of NAFLD and NASH. Other commonly prescribed drugs were saroglitazar (n=522, 85.7%), hepatoprotective agents (n=366, 60.1%), obeticholic acid (n=255, 41.9%), and pioglitazone (n=141, 23.2%; Table 5).

In this survey, of the 609 physicians, 525 (86.2%) reported prescribing vitamin E for treating NASH, and UDCA was prescribed by 341 (56%) physicians, while 26 (4.3%) physicians recommended alternative medicine or herbal preparations. Aside from pharmacotherapy, dietary and lifestyle modifications were also recommended to more than 30% of the patients with NAFLD.

Of the 609 physicians, 498 (81.8%) reported that they followed up with their patients with NAFLD every 3 months. To monitor the response of patients to pharmacotherapy, 558 (91.6%) physicians reported using liver function tests, while 477 (78.3%) preferred elastography, 234 (38.4%) used different scoring methods based on serum markers, and 188 (30.9%) used ultrasonography (Table 6).

Of the 609 physicians, most (n=347, 57%) referred to the American Association for Study of Liver Diseases (AASLD) guidelines, while 148 (24.3%) physicians referred to the Asian Pacific Association for the Study of the Liver (APASL) guidelines for diagnosing and managing patients with NAFLD. In terms of treatment barriers, most physicians (n=466, 76.5%) considered lack of patient awareness as a major barrier to managing NASH. Additionally, 303 (49.8%) physicians reported a lack of availability of effective drugs, and 264 (43.3%) reported the cost of evaluation and treatment as barriers (Table 7).

The drugs considered by physicians as potential future therapeutic options for the management of NAFLD and NASH included peroxisome proliferator–activated receptor α and γ agonists (n=348, 57.1%), glucagon-like peptide-1 (GLP-1) agonists (n=271, 44.5%), farnesoid X receptor agonists (n=262, 43%), and sodium-glucose cotransporter-2 inhibitors (n=219, 36%).

**Table 5:** Drugs commonly prescribed for the management of nonalcoholic fatty liver disease (NAFLD) and nonalcoholic steatohepatitis (NASH; N=609).

Commonly prescribed drugs for NAFLD and NASH management	Respondents, n (%)
Antioxidant vitamins	543 (89.2)
Saroglitazar	522 (85.7)
Hepatoprotective agents	366 (60.1)
Obeticholic acid	255 (41.9)
Pioglitazone	141 (23.2)

**Table 6:** Investigations preferred by physicians to monitor patients’ responses to pharmacotherapy (N=609).

Clinical investigations	Survey responses, n (%)
Liver function tests	558 (91.6)
Elastography	477 (78.3)
Different scoring based on serum markers (aspartate aminotransferase-to-platelet ratio index score and Fibrosis-4 score)	234 (38.4)
Ultrasonography	188 (30.9)
Liver biopsy	40 (6.6)
Magnetic resonance imaging	29 (4.8)

**Table 7:** Barriers to the management of nonalcoholic steatohepatitis (NASH; N=609).

Barriers in NASH management	Respondents, n (%)
Lack of awareness in patients	466 (76.5)
Lack of availability of effective drugs	303 (49.8)
Cost of evaluation and treatment	264 (43.3)
Time constraint	105 (17.2)
No barriers	9 (1.5)

## Discussion

### Principal Findings

This nationwide survey of 609 Indian gastroenterologists and hepatologists provides a comprehensive understanding of real-world practices in the diagnosis and management of NAFLD and NASH. The findings highlight that NAFLD accounts for a substantial proportion of liver disease cases seen in daily practice, with obesity and diabetes being the most frequently coexisting metabolic comorbidities, consistent with the well-documented pathophysiologic overlap between metabolic syndrome and NAFLD in Indian patients [1,2,4]. Notably, this survey was conducted between May 2023 and July 2023, before the official nomenclature change from NAFLD and NASH to MASLD and MASH was announced globally; therefore, the previous terminology was used throughout the questionnaire and data reporting to ensure consistency.

Although TE was the most frequently used diagnostic modality in our survey (reported by 558/609, 91.6% of the physicians), its diagnostic value lies primarily in the noninvasive assessment of liver fibrosis rather than the differentiation between simple steatosis and steatohepatitis (MASH). Current international guidelines from AASLD, European Association for the Study of the Liver (EASL), and APASL recommend an initial screening with simple serum-based fibrosis indices such as FIB-4 or the NAFLD fibrosis score, followed by TE as a secondary assessment in patients at intermediate or high risk [9,20,21]. The predominant reliance on TE observed in our cohort likely reflects its availability and routine use in tertiary centers, where most patients are referred cases from consulting physicians or secondary-care facilities. In such settings, TE serves as a valuable tool for fibrosis staging and risk stratification. However, optimizing its use in accordance with global stepwise algorithms—including initial screening with low-cost serum indices—would ensure more efficient resource use and enhance adherence to international best practices across all levels of care in India.

In contrast, pharmacological management patterns were dominated by saroglitazar (n=522, 85.7%) and vitamin E (n=525, 86.2%), and hepatoprotective agents such as UDCA and silymarin remained frequently prescribed despite limited supporting evidence [17,22]. These findings illustrate the diversity of real-world clinical approaches in India and highlight opportunities to further align everyday practice with evolving evidence and guideline-based recommendations.

### Comparison With Prior Work

Our findings are consistent with previously published Indian studies and surveys showing variability in NAFLD diagnosis and treatment pathways [3,5,17]. Similar reliance on TE and limited use of validated scoring systems was reported by Singh et al [6] and Duseja et al [9], emphasizing the need for broader adoption of noninvasive fibrosis indices for early risk stratification. Internationally, AASLD, EASL, and APASL recommend a tiered diagnostic pathway beginning with simple serum-based scores, followed by elastography or imaging only when advanced fibrosis is suspected [20,21].

The predominance of saroglitazar use (n=522, 85.7%) in this survey reflects its availability and regulatory approval for NAFLD in India. However, current global guidelines have not endorsed saroglitazar as a first-line therapy, and its approval outside India remains limited [15,17]. Although Indian real-world studies report biochemical and metabolic improvements, long-term histological and outcome data remain limited. Our survey did not capture details regarding cost, accessibility, or physician perceptions influencing prescription behavior; therefore, these findings should be interpreted as descriptive of current practice rather than indicative of comparative efficacy or cost-effectiveness. This finding underscores the need to examine economic and awareness factors shaping therapy choices in future research.

GLP-1 receptor agonists (RAs) such as semaglutide show histological improvement in NASH and metabolic benefits in global phase 3 trials [23,24]. When the DRIVE survey was conducted, injectable semaglutide was not approved in India; therefore, questions on its clinical use were not included. Instead, GLP-1 RAs were listed among potential future therapeutic options, for which only a small proportion of clinicians expressed interest. Given that semaglutide is still not approved for NASH in India and requires parenteral administration, widespread use is currently limited. Future studies should assess the real-world uptake, feasibility, and outcomes associated with GLP-1–based therapies in Indian clinical practice.

The term “hepatoprotective agents” in our questionnaire encompassed widely used drugs such as UDCA, silymarin, and antioxidant formulations. These agents are not recommended by AASLD, EASL, or INASL for NAFLD management due to the absence of histological benefit [9,21]. Their continued use by more than half of the surveyed clinicians (n=366, 60.1%) likely reflects therapeutic inertia, overreliance on empirical, perceived safety, and patient demand. However, this pattern must also be interpreted in the context of limited pharmacological options available in India during the survey period—till early 2025, saroglitazar remained the only approved agent for NASH, and no other globally validated drugs had received regulatory clearance. In the absence of alternative evidence-based therapies, clinicians may have continued to rely on hepatoprotective agents to provide symptomatic or biochemical improvement. These findings suggest opportunities to strengthen clinician education; wider dissemination of updated practice guidelines; and accelerated access to effective, evidence-based therapies to address the unmet clinical needs in NAFLD management.

Globally, there has been a paradigm shift in nomenclature from *NAFLD* and *NASH* to *MASLD* and *MASH* [7,8]. This change was formally endorsed in June 2023 by major international societies, including AASLD and EASL, reflecting a conceptual move away from alcohol exclusion criteria toward emphasizing metabolic dysfunction as the central driver of disease. The DRIVE survey was conducted before this global transition; therefore, it used NAFLD and NASH terminology, which has been retained in this study to maintain accuracy and continuity of the survey data.

In the INASL 2023 Guidance Paper, it was explicitly acknowledged that the recent nomenclature change “may create confusion not only among hepatologists but also among physicians and non-hepatologists who manage these patients or conduct research in this area” [9]. Accordingly, INASL continues to recommend the use of NAFLD and NASH terminology within India until uniform adoption of the MASLD and MASH framework is achieved.

In addition to metabolic drivers of steatotic liver disease, HCC may arise in patients with MASLD even in the absence of cirrhosis. Onzi et al [25] reported that a significant proportion of HCC cases in NAFLD occur in noncirrhotic livers, suggesting that hepatocarcinogenesis in MASLD involves metabolic and inflammatory pathways independent of advanced fibrosis. Soldera et al [26] further emphasized the need to integrate risk factors such as diabetes and obesity into HCC surveillance frameworks, highlighting the relevance of the Barcelona Clínic Liver Cancer protocol for risk-based screening. Given India’s rising burden of diabetes and obesity, these findings support reconsidering surveillance criteria to include selected patients with MASLD who are noncirrhotic at elevated risk for HCC.

### Strengths and Limitations

This survey has several notable strengths. First, it captured perspectives from a large, nationally representative cohort of 609 practicing gastroenterologists and hepatologists across diverse health care settings in India, providing a robust overview of real-world diagnostic and therapeutic approaches. Second, it used a structured, predefined questionnaire based on national and international clinical guidance, ensuring consistency and comparability of responses. Third, the survey included both hepatologists and gastroenterologists, reflecting multidisciplinary viewpoints and providing a realistic picture of current clinical practice. Fourth, because the survey was conducted immediately before the global transition from NAFLD and NASH to MASLD and MASH terminology, it provides a baseline on physician practices before the nomenclature change—information that will be valuable for future longitudinal comparisons.

However, several limitations merit mention. First, responses were self-reported and may be subject to recall or social-desirability bias. Second, as participation was voluntary and the total number of invitations sent was not recorded, representativeness and the response rate could not be assessed. Third, as this was a noncomparative, exploratory survey aimed at describing clinical practice patterns, only descriptive statistical analyses were conducted, and inferential statistics were not performed; hence, causal inferences cannot be drawn. Fourth, this survey reflects physician perceptions and may not fully represent patient-level practice data. Fifth, newer pharmacological agents and diagnostic technologies may have been underrepresented, given the survey’s cross-sectional design and timing before the MASLD nomenclature shift. Sixth, the survey did not include specific questions on HCC risk assessment or surveillance practices, an important area of growing relevance given the emerging evidence of HCC development in noncirrhotic MASLD. Finally, the survey did not include questions on drug affordability, availability, or rationale for therapy selection, which limits the interpretation of the observed preference for therapeutic options. Therefore, while the survey provides valuable insights into prevailing clinical practices, the findings should be interpreted cautiously and may not be fully generalizable to all health care settings, including primary care.

### Future Directions

Future research should explore longitudinal changes in diagnostic and treatment practices as the MASLD and MASH terminology and updated clinical frameworks become integrated into Indian hepatology practice. Strengthening implementation and adherence to existing India-specific consensus guidelines such as those issued by INASL [9] will be critical to ensure diagnostic accuracy and affordability, particularly by promoting the stepwise use of noninvasive scores (FIB-4 and NAFLD fibrosis scores) before TE. Educational interventions and continuous medical education programs can be instrumental in promoting rational pharmacotherapy and evidence-based management [18]. However, a major barrier in the Indian context remains the limited availability of approved, effective agents for NAFLD and NASH. Addressing this gap will require focused research and development on novel therapies—particularly those demonstrating antifibrotic effects—along with timely regulatory approval and improved access to such agents once available.

National liver societies should prioritize promoting evidence-based treatment practices and gradually phase out the use of hepatoprotective agents with limited clinical benefits, alongside structured dissemination of MASLD nomenclature and risk-adapted HCC surveillance even in noncirrhotic metabolic liver disease [7,26]. Establishing multicenter registries and real-world databases can further support health-economic evaluations and inform locally relevant guideline updates. Although this survey focused on Indian clinicians, its findings have wider international relevance, as the challenges identified—limited patient awareness leading to delayed diagnosis, few effective or approved treatment options, and discrepancies between drugs approved in India (eg, saroglitazar) and those endorsed internationally (eg, GLP-1 RAs)—mirror challenges faced by other low- and middle-income countries [13]. These insights can help inform global implementation strategies for MASLD management in diverse health care settings.

### Conclusions

This nationwide survey highlights considerable variability between prevailing clinical practices in India and international guidelines for the diagnosis and management of metabolic dysfunction–associated liver disease. While TE has become the most widely used noninvasive test, reliance on it without previous application of simple scores such as FIB-4 or the NAFLD fibrosis score indicates gaps in stepwise risk stratification. Saroglitazar remains the most commonly prescribed drug owing to its domestic approval and availability, but its long-term histological efficacy and cost-effectiveness require further evidence. The continued use of hepatoprotective agents such as UDCA and silymarin highlights the persistence of non–evidence-based treatments.

The transition from NAFLD and NASH to MASLD and MASH nomenclature provides an opportunity for India to harmonize diagnostic frameworks, enhance physician education, and modernize treatment algorithms. Future national efforts should focus on stepwise noninvasive staging, rational pharmacotherapy, risk-adapted surveillance for HCC—including in selected patients with MASLD who are noncirrhotic—and large-scale real-world registries to monitor outcomes. Harmonizing Indian clinical practice with evolving international standards is imperative to enhance diagnostic accuracy, consistency, and patient outcomes in metabolic dysfunction–associated liver disease.

## Appendix

Multimedia Appendix 1 Survey questionnaire form administered to the gastroenterologists and hepatologists.

Multimedia Appendix 2 CHERRIES guidelines.

## Abbreviations

AASLD: American Association for Study of Liver Diseases
APASL: Asian Pacific Association for the Study of the Liver
CHERRIES: Checklist for Reporting Results of Internet E-Surveys
DRIVE: disease perspective
EASL: European Association for the Study of the Liver
FIB-4: Fibrosis-4
GLP-1: glucagon-like peptide-1
HCC: hepatocellular carcinoma
INASL: Indian National Association for Study of the Liver
MASH: metabolic dysfunction–associated steatohepatitis
MASLD: metabolic dysfunction–associated steatotic liver disease
NAFLD: nonalcoholic fatty liver disease
NASH: nonalcoholic steatohepatitis
RA: receptor agonist
TE: transient elastography
UDCA: ursodeoxycholic acid

## Data Availability

The datasets generated or analyzed during this study are available from the corresponding author upon reasonable request.
