# Supplementary material for: Perspectives of Indian Gastroenterologists and Hepatologists on Nonalcoholic Fatty Liver Disease Diagnosis and Management: Insights From the Nationwide Web-Based Cross-Sectional DRIVE Survey
Source: Interact J Med Res. 2026 Mar 2;15:e75138. doi: 10.2196/75138 (PMC12993273; doi:10.2196/75138)
Supplement: Multimedia Appendix 1 [file ijmr_v15i1e75138_app1.docx]

## **Multimedia Appendix 1:** Survey questionnaire form administered to the gastroenterologists and hepatologists.

**Doctor’s details:**

Name:

Age:

Gender:

Years of clinical practice:

1. < 5 years b. 6-10 years c. 11-20 years d. >20 years

Predominant type of practice:

1. Multi-specialty hospital b. Clinic/ nursing home c. Government hospital/ medical college d. Mixed

**Disease perspective:**

1. What proportion of NAFLD cases do you see per month?
2. <25% b. 25-50% c. 50-75% d. >75%
3. Based on your experience what proportion of NAFLD patients have non-alcoholic steatohepatitis (NASH)?
4. <10% b. 10-20% c. 20-30% d. >30%
5. What percentage of your NASH patients undergo biopsy?
6. <5% b. 10-20% c. 20-30% d. >30%
7. At what stage of fibrosis most of your NAFLD patients first present to you?
8. Fibrosis stage 1
9. Fibrosis stage 2
10. Fibrosis stage 3
11. Fibrosis stage 4
12. Based on your clinical experience, approximately what percentage of NASH patients progress to cirrhosis over 5 years’ time?
13. <5% b. 5-10% c. 10-15% d.15-20% e. >20%
14. What percentages of total NAFLD patients coming to you are referred patients from other specialists?
15. 10-20% b. 20-40% c. 40-60% d. 60-80% e. >80%
16. From which of the following specialty you get the referral for NAFLD patients commonly?
17. Consulting physician
18. Endocrinologist
19. Cardiologist
20. Any other
21. How many percentages of your patients get incidentally diagnosed with NAFLD?
22. < 25% b. 25-50% c. 50-75% d. >75%
23. What do you do if you find NAFLD incidentally in your patient? (You can tick multiple options)
24. Investigate presence of metabolic alterations and the absence of other liver diseases
25. Assess the cardiovascular risk and investigate if the patient has a liver disease
26. Assess the risk of metabolic alterations and liver function with non-invasive scoring system in absence of other chronic liver disease
27. Investigate presence of alcohol consumption
28. Advise lifestyle modification and regular follow up
29. Initiate pharmacotherapy
30. Which of the following do you consider as risk factors for NAFLD? (You can tick multiple options)
31. Diabetes mellitus
32. Hypertension
33. Obesity
34. Polycystic ovary syndrome
35. Dyslipidemia
36. Sleep apnea
37. Hypothyroidism
38. Ischemic heart disease
39. What are the percentages of your NAFLD patients who have following co-morbidities?
40. Diabetes mellitus: a. 20-40% b. 40-60% c. 60-80% d. >80%
41. Obesity: a. 20-40% b. 40-60% c. 60-80% d. >80%
42. Dyslipidemia: a. 20-40% b. 40-60% c. 60-80% d. >80%
43. What percentage of your NAFLD patients require multispecialty management approach (for e.g. endocrinologist, diabetologists, cardiologist etc.) due to multiple co-morbidities?
44. <10% b. 10-30% c. 30-50% d. >50%
45. What proportion of lean NASH among all NASH patients do you diagnose per year?
46. 1-3% b. 3-5% c. 5-10% d. >10%
47. Do you recommend screening of family members of NAFLD diagnosed patients?

Yes No

1. What percentages of your NAFLD patients are symptomatic?
2. <10% b.11-30% c. 31-60% d. >60% e. Do not know
3. Which of the following symptoms are commonly reported by NAFLD patients? (You can tick multiple options)
4. Bloating b. weakness c. fatigue/ tiredness d. right upper quadrant pain e. A feeling of fulness f. Mention other symptoms if any.

**Diagnostic modalities:**

1. Which of the following investigations do you prefer for diagnosis of NAFLD? (You can tick multiple options)
2. MRI- PDFF
3. Transient elastography (Fibroscan/ fibrotouch)
4. MR elastography
5. CT scan
6. Liver biopsy
7. Kindly specify other investigations performed if any:
8. Which of the following additional data/ laboratory parameters do you routinely ask in NAFLD diagnosed patients? (You can tick multiple options)
9. Body mass index (BMI)
10. Waist circumference
11. Blood pressure measurement
12. Blood glucose levels
13. Homeostatic Model Assessment of Insulin Resistance (HOMA-IR)
14. Lipid profile
15. Which of the following scoring methods do you use for the assessment of severity of NAFLD? (You can tick multiple options)
16. BARD score
17. Fibrometer
18. NAFLD fibrosis score
19. APRI score
20. Fib-4
21. AST/ ALT ratio
22. None
23. When you prescribe a liver biopsy in patients with NAFLD, it is mainly with the intent to?
24. Distinguish NASH from steatosis: diagnostic purpose
25. Staging of fibrosis: prognostic purpose
26. Any other please mention: ______________

**Management strategies:**

1. How do you manage patients with NAFLD?
2. Dietary and lifestyle modification alone
3. Pharmacotherapy along with dietary and lifestyle modifications
4. In what percentages of your NAFLD and NASH patients do you prescribe a pharmacological therapy-
5. Percentage of NAFLD patients:

a. 11-20% b.21-40% c. 41-60% d.>60%

1. Percentage of NASH patients:

a. 21-40% b. 41-60% c.61-80% d. >80%

1. What percentages of your patients do you refer to dietician for dietary management?
2. <10% b. 11-20% c. 21-30% d. >30%
3. What is the daily alcohol consumption you allow for your NASH patients?
4. 0-gram b. 10-30gram
5. Which of the following drugs do you prescribe most commonly for management of NAFLD/ NASH? (Please tick up to three options)
6. Anti-oxidant vitamins
7. Hepato-protectant agents
8. Obeticholic acid
9. Saroglitazar
10. Pioglitazone
11. Which anti-oxidant vitamin do you prescribe most commonly in your NAFLD patients?
12. Vitamin E b. Vitamin D c. Vitamin A d. Vitamin C
13. Which hepato-protectant do you commonly use in NAFLD management?
14. Urso-deoxycholic acid b. Silymarin c. N-adenosyl methionine d. Any other, please mention
15. Details of drugs prescribed for NASH. (Please tick more than one options if needed as appropriate)

| Drug | Dose (in mg) | Duration in months |
| --- | --- | --- |
| Vitamin E | Mention range of dose used  ------------------------- | ☐3, ☐6 ☐12, ☐18, ☐24 |
| Urso-deoxycholic acid | Mention range of dose used  ------------------------ | ☐3, ☐6 ☐12, ☐18, ☐24 |
| Obeticholic acid | ☐5, ☐10 | ☐3, ☐6 ☐12, ☐18, ☐24 |
| Saroglitazar | ☐4 | ☐3, ☐6 ☐12, ☐18, ☐24 |
| Pioglitazone | Mention range of dose used  ----------------- | ☐3, ☐6 ☐12, ☐18, ☐24 |

1. Do you recommend alternative medicine/ herbal preparations in patients with NAFLD?

Yes No

1. How frequently do you follow up NAFLD/ NASH patients?
2. 3 months
3. 6 months
4. >6 months
5. How do you monitor the response to pharmacotherapy? (You can tick multiple options)
6. Liver function tests
7. USG
8. Elastography
9. MRI
10. Liver biopsy
11. Different scoring system based on serum markers e.g. APRI score, Fib-4
12. Which of the following guideline do you refer for diagnosis and management of patients with NAFLD?
13. American Association for the Study of Liver Diseases (AASLD)
14. European Association for the Study of the Liver (EASL)
15. Asian Pacific Association for the Study of the Liver (APASL)
16. Mention any other guidelines if followed ______________
17. Which of the following do you consider as barrier for the management of NASH? (You can tick multiple options)
18. Time constraint
19. Cost of evaluation and treatment
20. Lack of awareness in patients
21. Lack of availability of effective drugs
22. No barriers
23. Which of the following class of drug do you see as a potential class for future therapeutic option for management of NAFLD? (You can tick multiple options)
24. Thyroid hormone β-receptor agonist
25. Glucagon like peptide-1 (GLP-1) agonist
26. Dual Glucose-dependent insulinotropic polypeptide (GIP) and GLP-1 agonist
27. Peroxisome proliferator activated receptor (PPAR) α and γ agonist
28. Farnesoid X receptor (FXR) agonist
29. GIP and GLP-1 agonist
30. Sodium glucose cotransporter-2 (SGLT-2) inhibitor
31. Any other
