# Supplementary material for: Perspectives of Indian Gastroenterologists and Hepatologists on Nonalcoholic Fatty Liver Disease Diagnosis and Management: Insights From the Nationwide Web-Based Cross-Sectional DRIVE Survey
Source: Interact J Med Res. 2026 Mar 2;15:e75138. doi: 10.2196/75138 (PMC12993273; doi:10.2196/75138)
Supplement: Multimedia Appendix 2 [file ijmr_v15i1e75138_app2.docx]

**Multimedia Appendix - 2**

**CHERRIES checklist**

| **S. No.** | **Item** | **Description for DRIVE survey** | **Manuscript section** |
| --- | --- | --- | --- |
| 1 | Design | Cross-sectional, web-based questionnaire survey among gastroenterologists and hepatologists across India | Methods → *Participants and Procedures* |
| 2 | IRB approval and consent | Approved by Royal Pune Independent Ethics Committee, Pune, Maharashtra (Approval No. RPIEC110123, dated Jan 10, 2023). As this was a physician-based survey with anonymized data, patient-informed consent was not applicable | Methods → *Ethical Considerations* |
| 3 | Development and pretesting | The survey questionnaire (34 items) was developed by the study investigators based on national (INASL) and international (AASLD) clinical practice guidelines for NAFLD/NASH management and was reviewed by hepatology experts for relevance and clarity prior to deployment | Methods → *Survey Questionnaire* |
| 4 | Recruitment process and description of the sample having access to the questionnaire | Eligible participants were practicing gastroenterologists and hepatologists in India, who voluntarily agreed to participate in this survey | Methods → *Participants and Procedures* |
| 5 | Survey administration | The survey was hosted on a secure web platform (THB proprietary survey engine; HTTPS-encrypted). Responses were collected between May and July 2023 | Methods → *Participants and Procedures* |
| 6 | Participation rate (view rate, participation rate, completion rate) | Of ~1000 invitations, 609 participants completed the survey, and partial responses were not analyzed |  |
| 7 | Preventing multiple entries | Screening ensured one entry per respondent |  |
| 8 | Survey structure | 34 questions across three domains: disease perspectives (16), diagnostic modalities (4), management strategies (14). Mix of multiple-choice, Likert, and open-ended questions | Methods → *Survey Questionnaire* |
| 9 | Randomization of items or order | Unreported |  |
| 10 | Adaptive questioning | None |  |
| 11 | Number of items per page | Unreported |  |
| 12 | Mandatory/optional items | All questions were mandatory |  |
| 13 | Completion time | Unreported |  |
| 14 | Data analysis | Data exported into SPSS v16.0. Descriptive statistics (frequencies, percentages) were computed; open-ended responses analyzed thematically | Methods → *Statistical Analysis* |
| 15 | Handling of incomplete questionnaires | Only fully completed surveys (n = 609) were included in analysis | Methods → *Participants and Procedures* |
| 16 | Statistical corrections | Not applicable (purely descriptive analysis) | Methods → *Statistical Analysis* |
| 17 | Data security | Data stored on password-protected servers, accessible only to study investigators. All identifiers removed before analysis | Methods → *Ethical Considerations* |
| 18 | Incentives | No incentives provided |  |
| 19 | Accessibility | The survey was administered only to participating clinicians | Methods → *Participants and Procedures* |
| 20 | Reporting standard citation | This survey adheres to the CHERRIES (Checklist for Reporting Results of Internet E-Surveys) guidelines (Multimedia Appendix 2) | Methods → *Reporting Standards* |
